# The Relationship Between CEO Duality and Business Firms’ Performance: The Moderating Role of Firm Size and Corporate Social Responsibility

**DOI:** 10.3389/fpsyg.2021.669715

**Published:** 2021-12-30

**Authors:** Riaqa Mubeen, Dongping Han, Jaffar Abbas, Susana Álvarez-Otero, Muhammad Safdar Sial

**Affiliations:** ^1^School of Management, Harbin Institute of Technology (HIT), Harbin, China; ^2^Antai College of Economics and Management (ACEM), School of Media and Communication (SMC), Shanghai Jiao Tong University (SJTU), Shanghai, China; ^3^Department of Business Administration, Faculty of Economics and Business, University of Oviedo, Oviedo, Spain; ^4^Department of Management Sciences, COMSATS University Islamabad (CUI), Islamabad, Pakistan

**Keywords:** corporate social responsibility, CEO duality, firm size, board of directors, firm performance

## Abstract

This study focuses on exploring the relationship between chief executive officer (CEO) duality and firm performance. We focus on how the size and corporate social responsibility (CSR) of firms moderate this relationship. In terms of size, business organizations are of two types: small and large firms. This study uses datasets of listed Chinese business firms included in the China Stock Market and Accounting Research database. It employs a generalized method of moment’s technique to explore the connection between CEO duality and the performance of Chinese business firms through double mediation effects. Our empirical analysis showed that CEO duality has a significant negative relationship with firm performance. We also explored the moderating effects of firm size (small and large) and CSR practices on the relationship between CEO duality and improved performance of Chinese firms. Large firms and CSR practices showed significant and positive moderating effects on the relationship between CEO duality and firm performance. Conversely, with CEO duality, small firms showed a negative moderating influence on firm performance. This inclusive model provides valuable insights into how the dual role of the CEO of a firm affected the performance of Chinese firms through the moderating role of CSR practices and firm size for better business performance. The study offers empirical and theoretical contributions to the corporate governance literature. This research framework might help researchers in designing robust strategies to evaluate the effects on firm performance. Researchers may gain helpful insights using this methodology.

## Introduction

Chief executive officers’ dual roles (CEO duality) come from the board structure of leadership. The positions of CEO (chief executive officer) and board chairman are combined into a single role (Barroso et al., 2011), counteracting the powers of the board of directors (Pérez-Calero et al., 2016). This role provides CEOs with additional capabilities and strengths (Rajan and Zingales, 1995) but results in mixed outcomes for organizations (Jensen and Murphy, 1990; Baker and Hall, 2004). Two conflicting perspectives (based on agency and stewardship theories) underlie the relationship between CEO duality and business firm performance (Moeller et al., 2004; Singh et al., 2018). An argument based on agency theory is that a CEO with dual powers provides a more robust decision-making structure that results in higher firm performance (Baker and Hall, 2004; Youn et al., 2015). Conversely, a stewardship theory argument is that a CEO with dual positions/roles could take advantage of the dual role to pursue personal gains rather than firm benefit as the top priority (Brammer and Millington, 2006). Such disagreement on CEO duality and its influence on firm performance has led to continued research interest. However, the theoretical approach to this issue appears slightly unclear and contradictory (Barroso et al., 2011; Pérez-Calero et al., 2016). While some studies assert that CEO duality can negatively impact firm performance (Moeller et al., 2004), others suggest no significant impact (Freeman et al., 2010; Abbas et al., 2019b), and others provide evidence of a positive association (Javeed and Lefen, 2019).

Accordingly, with these inconsistencies suggesting the presence of moderating and intervening elements between CEO duality and business performance, researchers have urged for more studies on this issue (Torugsa et al., 2012; Guillet et al., 2013; Javeed and Lefen, 2019). Existing studies suggest that academics should consider moderating elements when investigating the effect of CEO duality on the decision-making of firms (Fosberg, 2004; Sheikh, 2018; Naseem et al., 2019). They recommend that task requirements alter the CEO and firm performance association when performance involves additional information concerning innovative and high-quality decisions. Studies have explored the influence of CEO duality on the performance of business firms through moderating effects of environmental context and firm size (Guillet et al., 2013). A previous study has indicated that firm size and environmental context played a moderating role in the relationship between CEO duality and business firm performance (Guillet et al., 2013; Li and Chen, 2018). Therefore, this study proposes a model to investigate how firm size and corporate social responsibility (CSR) moderate the relationship between CEO duality and business performance.

Note that firm size (small and large) is of considerable importance in firm performance and organizational management. Studies (Rajan and Zingales, 1995; George and Hall, 2004) have reported that the structure of debt and CEO managerial products increase with firm size. One study argued that the benefits of executives increase with firm size (Jensen and Murphy, 1990). Furthermore, firm size may constrain or facilitate the activities of business companies, namely, their decision-making and innovation processes (Youn et al., 2015). For example, larger firms with more advanced and well-organized structures can better respond to market changes and achieve desired performance. Another study noted that the activities of CEOs might vary by firm size because larger firms have a more significant market reputation and more resources than smaller ones (Moeller et al., 2004). A study (Singh et al., 2018) revealed that small businesses could generate more abnormal earnings. Besides, larger firms are more skilled and, hence, can produce new products and achieve desired goals. This study focuses on exploring the connection between CEO duality and the performance of Chinese firms. It analyzes how firm size and CSR moderate this relationship to attain better performance. CSR is another critical factor affecting the decision-making processes of business managers and influencing organizational performance (Freeman, 2010). Business firms can contribute to social responsibility and social good through their respective stakeholders, such as governments, consumers, workers, and shareholders. Studies have reported that investors and various stakeholders need to perceive CSR firms positively. Thus, CEOs use CSR to solve agency problems between shareholders and managers (Javeed and Lefen, 2019).

Business managers play a vital role in improving the performance of their firms by enhancing CSR practices and building a solid relationship with shareholders (Torugsa et al., 2012). In addition, a CEO can solve the issues of shareholders by engaging them in CSR activities and raising their wealth and the profits of business firms (Blacconiere and Patten, 1994). CSR policies help managers to gain competitive advantages and enhance firm values (Youn et al., 2015). Studies in the literature have reported that CEO duality can positively affect firm performance and CSR (Brown and Forster, 2013). Correspondingly, CSR is assessed as the basis of the competitive advantages of firms and means of increasing firm value (Popescu, 2019). This study concentrates on exploring firm size (small/large) and CSR practices as moderating effects on the relationship between CEO duality and the performance of Chinese firms No study has so far explored the combined effects of CSR activities and firm size (small and large) on the relationship between CEO duality and the business performance of Chinese firms. This research study aims to examine the role of CEO duality in the performance of Chinese firms through the moderating effects of CSR practices and firm size.

The empirical analysis of this study has demonstrated that CEO duality has a significant negative relationship with the performance of Chinese firms. The study has observed the moderating effects of firm size (small and large) and CSR practices on the relationship between CEO duality and the performance of Chinese firms. Hence, large business companies/firms and CSR significantly and positively moderate the relationship between CEO duality and the performance of Chinese firms. In contrast, small firms indicate a negative moderating influence of CEO duality on the performance of firms. This inclusive model offers valuable insight into the dual role of the CEO of a Chinese firm in achieving better business performance through the moderating effects of CSR practices and firm size. The study offers empirical and theoretical contributions to corporate governance literature. Our findings provide evidence that businesses can benefit by adopting CEO duality to attain better business performance. This research model has filled potential gaps in the literature with a novel framework in the context of an emerging economy. This study has discussed the main components of the organizational structure, such as firm size (small and large) and CSR.

This research model theoretically and empirically contributes to experiential studies exploring the influence of CEO duality on the performance of Chinese firms in the following manner: first, we examine the context of developing economies, because their organizational structure is distinctive from that of developed world economies. Second, no study has, so far, examined the moderating role of firm size (small and large) and CSR activities in the relationship between CEO duality and better firm performance. Hence, this is the first study to discover this gap in the literature and contributes to filling the gap in the context of a developing economy. Third, our research model investigates the moderation effect of CSR and firm size on the relation between Chinese firm CEO’s duality and better business performance in the context of the emerging Chinese economy. Fourth, this study analyzes the relationship of shareholders of Chinese firms, as this might benefit them enormously by developing internal organizational systems. This study presents a comprehensive empirical analysis of how the duality of a company CEO can result in better firm performance through the moderating effects of firm size (small and large) and CSR practices.

There are numerous reasons to select China for this study. First, China is the largest developing market and its economy is the second-largest in the world, showing a different atmosphere from established countries. Second, because of the inefficiency of financial markets, significant government involvement, and weak legal institutions, the organizational structure of emerging nations differs from that of developed ones (Saeed and Athreye, 2014). Diverse corporate governance methods may influence the effectiveness of CEO duality on company performance in developing nations. Third, current research suggests that this investigation examined this connection in the setting of a developing country with excellent corporate governance (Naseem et al., 2019). As a result, China is the most prominent and sizeable emerging economy with a well-developed governance system. It also provides an interesting environment for refining and testing prior organizational theories (Peng et al., 2010). Market participants and the government establish and review the Chinese corporate governance mechanism. The China Securities Regulatory Commission (CSRC) introduced changes in improving governance structure on top priority (Li and Chen, 2018). Chinese business organizations have steadily implemented corporate governance frameworks in response to market expansion and incorporated multiple measures for deciding for the powers of CEOs, board of directors, and executives (Conyon and He, 2011). These initiatives have helped in strengthening the governance structure of Chinese business organizations. These actions helped Chinese firms in implementing corporate governance reforms. Similarly, the literature has evidenced a substantial growth in the dual roles of CEOs. Besides improving the governance structure of firms, China has focused on developing a business culture that is useful to contribute to other developing countries as well. These measures have encouraged business firms to compete in the business industry (Su et al., 2013). In emerging countries, however, this link is still somewhat restricted. As a result of its large economy and extensive work on corporate governance, China is a desirable economy for this research.

This study presents an empirical analysis of the power of the duality of a CEO of a Chinese firm in achieving good business performance. The study is structured as follows: following the study pattern, section “Introduction” presents the Introduction. Section “Literature Review And Hypotheses” presents the framework and a literature review and develops the study hypotheses. Section “Research Methodology” presents the study sample and research methodology. The findings of the study are presented in section “Results,” while section “Discussion” concludes the study with its limitations and future directions.

## Literature Review and Hypotheses

### Chief Executive Officer Duality and Performance of Chinese Business Firms

Academia and researchers have debated the relationship between CEO duality and improved firm performance. Two theoretical perspectives primarily present the influence of the dual roles of a CEO of a firm on business performance in terms of the agency and stewardship theories. Stewardship theorists emphasize the positive effect of the dual role of a CEO on better firm performance (Freeman and Hasnaoui, 2011; Abbas et al., 2019b). Scholars supporting the stewardship theory explained that CEO duality could enhance the productivity of business firms by maximizing shareholder interests. The dual role of a CEO encourages administrative efficiency, improves communication, and provides a flexible management arrangement, shielding the interests of a firm through a better business organization (Desai et al., 2003). Business firms with entrepreneurial networks (Abbas et al., 2019c) innovation, knowledge sharing, and social media use (Abbas et al., 2019a) can achieve better sales and profitability. These can help firms improve their business performance (Hussain et al., 2019, 2021).

According to stewardship theorists, “top business firms’ managers have desired to become outstanding agents of their business resources” (Donaldson and Davis, 1991). Senior executives try to raise the profit of their business firms by holding dual positions in a business organization and decreasing agency expenses (Beasley, 1996). In addition to business governance perception, senior management can increase the expertise of executives and the value of shareholders through the knowledge of CEOs of firms of strategic procedures and challenges (Jensen and Heckling, 1995; Bhagat and Black, 2001; Iyengar and Zampelli, 2009). Thus, opinions favoring this theory endorse a positive influence of firm CEO duality on business performance. Conversely, agency theorists highlight the adverse impacts of CEO duality on business performance as the dual position of a CEO may pursue personal benefit rather than the profitability of a firm (Fama and Jensen, 1983). Thus, logically, CEO duality can lead to agency issues between stockholders and business managers. Moreover, CEOs can take advantage of their dual role in a weak structure and assign closer individuals executive positions through CEO directives (Faleye, 2007). These findings suggest that the dual role of CEOs can cause deterioration in the business monitoring structure and lead to diversity in firm performance (Krause, 2017).

The literature presents ambiguous and contradictory arguments supporting the agency and stewardship theories. The empirical literature related to the relationship between CEO duality and business performance is primarily indecisive. Previous studies have offered mixed results, with some robust studies unable to prove a strong relationship between CEO duality effects and better business performance (Duru et al., 2016; Javeed et al., 2020). In contrast, some studies have identified a positive CEO dual role impact on firm performance, while others have reported a negative CEO dual role influence on firm performance. Besides, some studies have found a positive connection between the duality of CEOs of firms and business performance based on the stewardship theory (Donaldson and Davis, 1991; Guillet et al., 2013; Wang et al., 2014; Nekhili et al., 2018). An earlier study examined the influence of CEO duality on better firm performance in the context of European business firms (Pham and Pham, 2020). It identified a positive effect of the CEO’s dual role on firm performance, supporting the stewardship theory (Guillet et al., 2013). Agency theory supporters acknowledged the negative influence of CEO duality on firm performance as the CEO could use the resources of the firm for personal interests. A previous study has investigated this relationship in United States industries and reveals a significant and negative relationship between CEO duality and business firm performance (Tang, 2017). Another study focused on exploring the impact of CEO duality on firm performance by examining datasets from Pakistan, an emerging economy, to find adverse effects on business performance (Naseem et al., 2019). Scholars reported a negative influence of CEO duality when using different study variables to measure business performance, such as return on equity (ROE) of firms, return on investment (ROI), stockholder return, and return on assets (ROA) (Guillet et al., 2013; Yang and Zhao, 2014; Tang, 2017; Naseem et al., 2019; Naseem, 2020). Agency theory advocates argue that a single governance structure might help in decisions that increase the positive effect of CEO duality on various shareholder expenses that lead to conflicts of interests between business managers and multiple shareholders (Iyengar and Zampelli, 2009). However, some studies could not identify any significant and positive CEO duality effect on firm performance (Kang and Zardkoohi, 2005; Iyengar and Zampelli, 2009). Conversely, most of the studies in the literature provide evidence that CEO duality has a negative impact on firm performance, ultimately supporting the agency theory (Wijethilake and Ekanayake, 2019). Hence, from the study findings mentioned above, we posit the following hypothesis:

H1: CEO duality has a negative influence on the performance of Chinese firms.

### Corporate Social Responsibility Moderates the Relationship Between Chief Executive Officer Duality and the Performance of Chinese Firms

Studies have suggested that the values and objectives of executives influence the CSR practices of a firm to attain better firm performance. CSR practices affect the decision-making processes of business managers. CSR practices of firms refer to their business activities from ethical and economic perspectives and reflect their economic profit and public welfare approach (Freeman and Hasnaoui, 2011). CSR describes activities in society and is defined as an obligation of groups and firms to decrease or eliminate unsafe corporate practices done in the wrong ways (Tarrow, 2001). The relationship between CSR and corporate governance has been examined in numerous studies. The CSR practices of firms establish business accountability internally and externally and reflect their profits (Chen and Hung-Baesecke, 2014). Investing in CSR activities can help firms to enhance their reputation and relationship with stakeholders (Chen and Hung-Baesecke, 2014; Tang et al., 2021).

Stakeholders can be classified into two groups: investing stakeholders (shareholders) and non-investing stakeholders (environment, local community, employees, customers, and suppliers). Additionally, CSR is said to increase firm value by considering all stakeholders equally and protecting their interests. A firm can decrease its risks and costs by establishing a proper relationship with its stakeholders. Competitive benefits may arise, but a worthy association with all shareholders always positively affects firm values (Harjoto and Jo, 2011). Overall, firm performance is positively related to CSR practices (Rehman et al., 2015). A firm with CSR activities that enhance stakeholder confidence will ultimately lead to higher firm performance (Adams, 2002). The reason for supporting CSR policies is that it causes firms to be accountable to the public and demonstrate their loyalty to society using CSR as a valuable tool (Adams, 2002). Second, shareholders usually assume that optimistic firms engage in CSR activities (Freeman and Hasnaoui, 2011). Some studies support the argument that enhancing environmental activities can improve firm performance (Adams, 2002; Casado-Díaz et al., 2014; Abbas et al., 2019b).

Chief executive officers are highly involved with organizational strategies concerning CSR activities, and this can lead to higher profits. Additionally, CSR revolves around solving issues between the CEO and shareholders in diverse matters (Wang and Chen, 2017). CEOs can use CSR to measure their association with shareholders and maintain a positive relationship (Chen and Hung-Baesecke, 2014; Shah et al., 2021). Thus, managers may want to maximize their long-term profits through improved connection with stakeholders, enhanced CSR practices, and better societal trust (Godos-Díez et al., 2011). However, CSR can be considered a shareholder expense that leads to lower firm performance, because executives invest in CSR activities for personal benefit (Barnett, 2007). Studies have shown that agency costs can be decreased through CSR practices and released to stakeholders (Jensen, 2002). Stakeholder representatives have also stated that CSR generates balanced wealth for shareholders and motivates other investors to participate in CSR firms. Studies have shown that firm performance is positively related to CSR. Furthermore, the CEO of a firm can use CSR to solve the issues of stakeholders and increase their wealth and firm profits (Jensen, 2002; Sen et al., 2006). We propose the following hypothesis by summarizing the above studies:

H2: CSR practices positively moderate the relationship between CEO duality and the performance of Chinese firms.

### Firm Size Moderates the Relationship Between Chief Executive Officer Duality and the Performance of Chinese Firms

Empirical and experimental studies related to the influence of CEO duality on Chinese enterprises/firms have produced mixed results (Tang, 2017; Singh et al., 2018; Naseem et al., 2020). However, not much evidence is available on why these results differ in the literature. A previous study has shown that some organizational factors may not allow firms to benefit from CEO duality (Naseem et al., 2020). It suggests that the effect of CEO duality on business performance might depend on some particular government environment; the contingency theory identified firm size as an organizational factor (Li and Chen, 2018). The literature has documented and recognized some moderating mechanisms that might help or limit business activities to achieve their desired objectives, such as interests of managers, firm improvement, and decision-making (Damanpour, 2010; Zona et al., 2013; Li and Chen, 2018). Even though scholars consider firm size (small and large) a critical study variable related to corporate finance studies, several studies consider it a control variable solely for exploring the connection between the duality of enterprise CEOs and firm performance (Li and Chen, 2018; Naseem et al., 2019). This study tries to identify whether firm size (small and large) moderates the relationship between CEO duality and business performance. This study also aimed to observe whether CEO duality improves or creates constraints for better outcomes. Hence, our model uses firm size (small and large) as a regulatory factor to explain why the empirical results on the relationship between CEO duality and company performance have been reported to be contradictory. From a theoretical perspective, small and large firms have different organizational structures (Li and Chen, 2018). For instance, large businesses have better financial resources than small organizations, as big organizations have extra financing opportunities to develop their business. A study has argued that large organizations would obtain more support because of their progress (Ezeoha, 2008). From another point of view, banks are always more eager to provide loans to more creditworthy business firms. The agency theory supporters claim that an organization would grow and try to cross state borders and become a significant business leader as its business develops. It would gain the capability to fulfill the goals of its shareholders and reduce its agency costs (Jensen, 2002; Maladjian and El Khoury, 2014; Mui and Mustapha, 2016; Raza and Karim, 2016; Hashmi et al., 2020). Besides, firm size plays a dynamic role in introducing new products or services to a firm. Large firms can quickly expand their business owing to their better resources as compared with smaller organizations. Small companies may be proficient in the construction and operative area where they are working (Hashmi et al., 2020). Accordingly, large business firms and corporations are more prominent in business activities. Their business operations may also reflect more noticeable and significant processes to produce more products, ensuring greater volumes of sales and profitability (Lee et al., 2014). The higher revenue level of business companies can lead to more income and an increase in profitability. It will enable them to produce more profits from the investment of stakeholders and their equity.

However, larger firms have more resources and a fair market reputation than smaller ones. They are more skilled to introduce new products and achieve desired goals (Rajan and Zingales, 1995; Moeller et al., 2004). In general, larger firms are more advanced and well-organized to respond to market changes. They can react quickly to shareholder pressures and work for shareholder interests rather than the private benefits of CEOs (Chandran and Rasiah, 2013). These firms have more assets and a well-organized structure and can compete in a competitive market and increase their profits (Jensen and Murphy, 1990). These arguments indicate that CEO duality helps larger firms perform better because of their organizational structure, good market reputation, and high market share (Weyzig, 2014).

Scholars have tried to study the effect of firm size on the profitability of business companies with different results ranging from negative, positive, or weak in terms of the selected variables. Previous research has reported a positive association between firm size and profits (Ibhagui and Olokoyo, 2018). One study analyzed the business performance of Greek firms over the period 1995–1999 (Papadogonas, 2007). This study divided Greek business firms into classes and employed regression analysis to draw the desired results. The results indicated that firm size affected the productivity and profitability of all categories of business companies. Studies have also examined the role of firm size by employing a fixed-effects model on a sample of over 7,000 publicly held United States firms (Lee, 2009). The research findings indicated that the size of the business firms played an indispensable role in enhancing sales and profitability (Amato and Burson, 2007). One study tested the relationship between profitability and firm size of companies operating in the financial services sector. Another study examined the nature of the relationship between firm size and profitability by employing financial and economic data (Andries and Faems, 2013). A study recognized a significant dissimilarity in profitability among medium, small, and large corporations (Ibhagui and Olokoyo, 2018). The investigation of firm size on the relationship between CEO duality and the performance of business firms assumes that the activities of executives and firm performance vary by firm size (Moeller et al., 2004; Dang et al., 2018; Sarfraz et al., 2020). Even though theoretical recommendations described the role of a CEO as changing over time with the size of other firms (Dang et al., 2018), these suggestions lead to possible pressure between firm size and CEO duality and are linked to firm performance (McWilliams et al., 2006). From the above debate, our model theorizes that firm size is a powerful mechanism to examine how CEO duality can increase or improve the performance of business firms (Yang and Zhao, 2014). The results of this study indicate why and how a selected variable affects other variables (Baron and Kenny, 1986). From this debate, we posit the following hypotheses:

H3: Firm size (small and large) moderates the relationship between CEO duality and the performance of Chinese firms.
H3 (a): There is a positive relationship between CEO duality and the performance of large-sized Chinese firms.
H3 (b): There is a negative relationship between CEO duality and the performance of small-sized Chinese firms.

### Model of a Proposed Research Study

**FIGURE 1:**
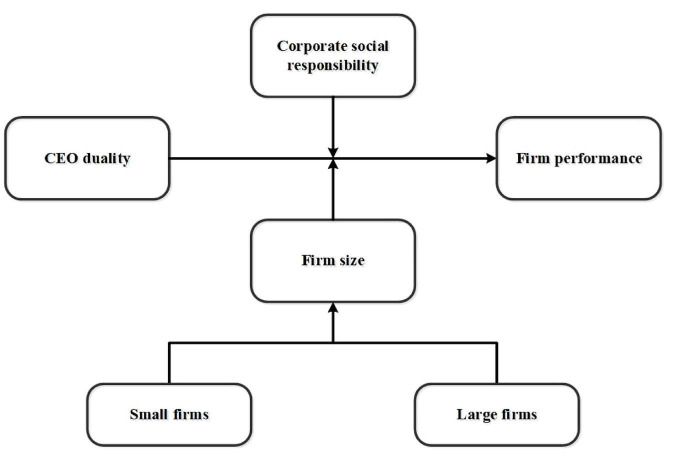
Theoretical framework with selected variables of this proposed study: CEO duality (independent variable, IV), the performance of Chinese firms (dependent variable, DV), and firm size (small and large) and corporate social responsibility (CSR) practices (moderating variables, MV).

This present proposed study model primarily focused on examining the relationship between CEO duality role and sustainable business performance of Chinese firms. This study explored how firm size and CSR practices moderate the relationship between CEO duality and the performance of Chinese business firms. This proposed study has incorporated four factors into this designed model. In this proposed research model, the duality of CEO is the independent variable, and this study incorporates the performance of Chinese firms as the dependent variable. CSR practices and firms’ size (small and large) are the moderating variables. The study applied a generalized method of moments (GMM) model to investigate the effects of CEO duality on business performance by taking advantage of previous research (Singh et al., 2018). Figure 1 demonstrates the theoretical framework of our proposed model after a detailed review of the scientific literature. See Figure 1, which describes the study model explained below.

## Research Methodology

### Sample and Data Collection

The primary datasets used in this research study are from the China Stock Market and Accounting Research (CSMAR) database, which contains the data of A-share listed companies and offers extensive details of financial data of Chinese firms (Mahmood et al., 2019). The initial sample consisted of listed Chinese firms in the manufacturing sector. This study employed a sample based on the 2012–2017 panel data because information related to the influence of CEO duality on business performance was not available and omitted. The datasets in the study are summarized as follows: (1) all companies with missing data were included, and then businesses with missing CEO duality and CSR information were removed. (2) The data of organizations that altered their administrative management structure were excluded, such as CEO single and dual positions (CEO and chair for the board of director positions). (3) Finally, a sample of 417 companies with 2,502 observations was selected for 6 years. As the biggest developing economy, China shows fast economic progress and great success in the emerging economy (Mamirkulova et al., 2020). Moreover, market participants and government authorities developed the governance structure of Chinese firms. For instance, an increasing number of firms in China have been considering CSR activities as a social good (Mubeen et al., 2020). A distinctive governance structure and quick stakeholder response make China an exciting research setting. Many scholars examine how CEO duality influences the performance of business firms in China.

### Variable Measurement

#### Assessing the Performance of Chinese Firms

Corporate governance scholars critically measure the performance of business firms, but previous studies have found no evidence of any inimitable methods to evaluate firm output. Studies have predominantly employed existing tools, such as ROA, ROI, ROE, and earnings per share (EPR) of firms, and Tobin’s Q to examine business firm performance (Javeed et al., 2020). Thus, corporate governance and accounting-based measurement studies measure firm performance best, because they reflect the ability of executives in firm efficiency and profits (Javeed et al., 2020). Therefore, this study employs ROE and ROI to observe business performance measures rather than other procedures, because these show the efficiency of management efficiency in the resources and production of firms.

#### Control Variables and Independent Variable (Chief Executive Officer Duality) of the Study

This study chose CEO duality as a primary independent variable to examine its effects on the business performance of Chinese firms. This variable is coded 1 for the CEO dual position, for instance, the chair position of the board of directors, and zero otherwise (Wang et al., 2019). CEO duality can have a negative effect on firm performance. This study also tested the control variables, such as total assets, growth opportunities, and the advantage of the firm. Mahmood et al. (2019) measured the leverage of firms as total liabilities to total assets ratio (Mahmood et al., 2019). Similarly, this study used growth opportunities as the variable in sales volume that affects the total revenue of a business. The tangibility of the assets of firms was calculated as the ratio of the equipment, plant, and properties of firms to their incremental revenue (Li et al., 2018).

#### Moderating Variables

Firm size is the most crucial variable used to measure the effect of CEO duality on business performance. Primarily, corporate finance studies use firm size as the control variable to assess the performance of business firms. However, this study identified a potential gap in the literature. Studies have suggested exploring the moderating influence of firm size (small and large) on the relationship between CEO duality and business performance because reasons for CEO duality in large and small businesses could be different (Guillet et al., 2013; Wang et al., 2019). This study argues that the influence of CEO duality on business performance would differ depending on the size of firms. This study addresses this gap in the literature by incorporating firm size (small and large) as a moderator and empirically analyzing the relationship between CEO duality and the performance of Chinese firms.

Accordingly, we found that large business firms might be more profitable and active in developing worldwide operations (Fiegenbaum and Karnani, 1991; Hardwick and Adams, 1999; Dillard et al., 2010; Chandran and Rasiah, 2013). Studies used different methods to measure the firm size in the corporate governance literature, such as total sales, natural log of total assets, and market equity assets. This study took firm size (small/large) as a moderator, evaluated its effect using the log of total sales volume, and followed the survey method (Dang et al., 2018). Here, the average firm size that we calculated by sales volume was 10.09. We then categorized the firms into two types by firm size: those more extensive than the average value were classified as large firms, and those below the average value were categorized as small firms. See Table 1 below that shows useful studies on the topic.

**TABLE 1:** Some critical studies on the chief executive officer (CEO) duality, firm size, corporate social responsibility (CSR), and firm performance.

Authors	Dependent/independent (Variables)	Country	Relationships	Method
Tang (2017)	Total shareholder return and CEO duality	Canada	Negative relationship	GEE
Duru et al. (2016)	Performance variables (ROA, ROE, ROS) and CEO duality	United States	Negative relationship	System GMM
(Akram et al., 2020)	CSR and firm growth	Pakistan	Positive relationship	2SLS and GMM
(Naseem et al., 2020)	Tobin’s Q and ROA	Pakistan	Negative	Fixed effects Model
(Yang and Zhao, 2014)	Tobin’s Q, ROA, and ROE with CEO duality	United States	Positive relationship	Baseline model
(Pham et al., 2015)	Performance variables (ROA, ROE, and Tobin’s Q) with CEO duality	Vietnam	Positive relationship	Two-stage least squares
(Guillet et al., 2013)	Performance variables (ROA and Tobin’s Q) with CEO duality	United States	Positive relationship	Two-way random-effects
(Iyengar and Zampelli, 2009)	Performance variables (ROA, MARKET, and Tobin’s Q) with CEO duality	United States	No significant relationship	Two-step treatment effects
Cornett et al. (2008)	ROA and CEO duality	United States	Negative and positive relations	Panel data regression analysis
(Elsayed, 2007)	Performance variables (ROA and Tobin’s Q) with CEO duality	Egypt	No significant relationship	LAV regression
(Al Farooque et al., 2007)	The market value of a firm with CEO duality	Bangladesh	No relationship	OLS and 2OLS

*Table 1 summarizes useful studies related to this proposed framework.*

Studies have suggested moderating mechanisms to study the impact of CEO duality on business firm performance. Accordingly, we considered CSR activities as the potential moderating variable to explore the effect of CEO duality on the business performance of firms (Guillet et al., 2013). Previous studies have used CSR practice indicators to evaluate similar variables, for instance, state rights, various stakeholder rights, and human and community rights. A survey study employed Carroll’s charter for research (Carroll, 1979) to assess the effects of CSR activities on firm performance (Asrar-ul-Haq et al., 2017). An earlier study in China investigated CSR indexes by considering 63 aspects, such as various stakeholder rights, human rights, and labor force rights, to observe proper operations (Han et al., 2018). Studies have explained that United States businesses established CSR indexes based on seven indicators: community, environment, human rights, relationships with employees, products, top management diversity, and governance (Blasi et al., 2018). Another study established a CSR index based on Shanghai Stock Exchange procedures, such as social contributions value (SCV) per share and its influence on the business performance of Chinese firms (Feng et al., 2018; Javeed and Lefen, 2019). Accordingly, this study investigated CSR practices as an SCV contribution to the per-share value index and social good, as suggested in the literature (Javeed and Lefen, 2019). The contribution value per share (SVR) index includes all necessary social components, namely, EPS, creating value for stakeholders, society, employees of firms, and reducing environmental pollution as a social expense (Feng et al., 2018). See Table 2 that shows the measures of all chosen variables in this research model.

**TABLE 2:** Measurement of variables.

Variables name	Measures
**Dependent variables**	
ROI (Returns on the investment)	Net profit divided by invested capital
ROE (Returns on equity)	Operating income divided by shareholders’ equity
**Independent variables**	
CEO duality	It has been coded as “1” for CEO’s who hold a chairman position for board of director, and coded “0” for other variables
Firm size	Log of firms’ total sales
Corporate social responsibility (CSR)	This study applied an index to CSR (Corporate social responsibility) as the firms’ social contributions per share (SCV), by following a past study (Feng et al., 2018).
Growth opportunities	It indicates a change in firms’ sales revenue of doing business activities.
Leverage	Leverage shows firms total liabilities and then divide it by total assets ratio
Asset tangibility	The ratio of plant, equipment, and property related to total revenue of a firm

*Variables detailed information and coding.*

### Empirical Approach

This study employed the GMM based on the support of existing studies that are based on some logical factors (Singh et al., 2018). First, we incorporated a panel dataset to examine the relationship between CEO duality and firm performance through the moderating role of firm size and CSR. Previous studies have shown that these endogeneity problems exist typically in panel datasets to produce biased and unreliable results (Javeed and Lefen, 2019). According to econometrics, if a variable has endogenous issues in the proposed model, scholars will suggest helpful ways to deal with them (Tien et al., 2013; Javeed et al., 2020).

Second, in a regression model, when there is a correlation between error terms, variables face endogeneity issues. In addition, when CEO decisions are correlated to error terms because of potential factors, these problems may arise from automatic regressions with missing variables, autocorrelation errors, and measurement errors (Adams et al., 2010). Therefore, these endogenous issues need to be controlled in CEO duality analysis.

Third, studies related to CEO duality positions advocate that CEO duality exists in potential endogeneity issues (Guillet et al., 2013). For instance, the appointments of CEOs and CEO chairs are endogenous (Nekhili et al., 2018). The probable effect of this on the performance of the CEO and CEO chair might determine the characteristics of firms that influence the CEO role and firm performance. Studies have also specified that the single leadership structure of a company is primarily endogenous and that the CEO dual position and CEO chairman role are related to some unobserved company characteristics, which could lead to endogenous problems (Kang and Zardkoohi, 2005; Li and Chen, 2018). Nevertheless, this study finds a positive relationship between endogeneity and unobservable firm characteristics.

Fourth, forming a panel dataset places limitations on the ordinary least squares (OLS) model, because it leads to biased estimations and could be inefficient because of unobserved heterogeneity (Wooldridge, 2002; Duru et al., 2016; Bae et al., 2018; Fralich and Fan, 2018; Javeed et al., 2020). Accordingly, it is particularly essential to control for endogenous problems in CEO duality analysis. Consequently, this study applied the GMM approach to explore the relationship between CEO duality and company performance to solve this endogenous problem. The findings of this study are in line with the literature (Singh et al., 2018).

Several econometric procedures are available to manage endogeneity issues. For instance, random effects and fixed-effect models, control variables, instrumental and lagged dependent variables, and the GMM model help control such issues (Li, 2016). Among all the analytical panel data techniques, the GMM model is the best approach with the highest power to deal with endogenous issues (Li, 2016). Because of its higher ability to deal with endogeneity, the preferred technique for this study is the GMM model. Previous studies have recommended the use of this model (Arellano and Bond, 1991). Within a company, the GMM model allows for autocorrelation and heteroscedasticity. The reliability of this model depends on instrumental validity. This study has described the details of the GMM model used, with suitable citations to support it. This model applied GMM, which allows testing for heteroscedasticity and autocorrelation of the chosen framework. The consistency of the GMM model depends on instrumental validity. Moreover, the GMM model is a more convenient and reliable approach than other techniques, as it has extreme effects to examine coefficient correction (Zou et al., 2015; Lin et al., 2019; Javeed et al., 2020, 2021; Banto and Monsia, 2021; Mittal and Garg, 2021). This study has incorporated the statistical software STATA for analysis purposes. It has tested a direct and moderating impact using the STATA software to confirm the significance of a test significance and satisfactory level. Tables 4, 5 present test results that show appropriate levels. Here, “*”indicates the significance level in this study model [significance levels: ^***^p < 0.01, ^**^p < 0.05, and *p < 0.1]. Accordingly, the GMM model was employed in this study, as recommended by numerous past studies, and our results are consistent with the literature findings (Li and Chen, 2018; Matemilola et al., 2018; Singh et al., 2018; Madaleno and Vieira, 2020; Boakye et al., 2021; Mittal and Garg, 2021).

**TABLE 3:** Variance inflation factor analysis and descriptive statistics.

Items	Panel A			Panel B	
Variable	Obs	Mean	Std. Dev.	VIF	1/VIF
ROI	2,502	0.340	0.605	1.33	0.750
ROE	2,502	0.461	0.985	1.40	0.712
Firm Size	2,502	10.09	0.570	2.33	0.43
CEO Duality	2,502	0.258	0.437	1.02	0.984
Growth	2,502	0.139	0.575	1.08	0.922
Leverage	2,502	0.088	0.094	1.26	0.794
Asset tangibility	2,502	3.00	5.87	2.20	0.453
CSR	2,502	1.950	1.76	1.04	0.96
VIF mean					1.49

*Std. Dev., standard deviation (SD); VIF, variance inflation factor.*

**TABLE 4:** Chief executive officer (CEO) duality and firm performance with the moderating impact of corporate social responsibility (CSR) practices.

	Model 1 (ROI)	Model 2 (ROE)	ROI (3)	ROE (4)
CEO Duality	−0.534** (0.261)	−1.21** (0.699)	1.521*** (0.7251)	1.01** (0.560)
CSR			0.198 (0.279)	0.289 (0.146)**
CSR*CEO duality			0.924** (0.492)	0.588*** (0.229)
Leverage	−5.44*** (1.25)	−6.08*** (2.13)	−6.86** (3.15)	−5.62*** (1.91)
Growth	0.068** (0.041)	−0.112 (0.148)	3.021** (1.78)	1.949* (1.213)
Firm size	2.73*** (0.558)	4.65*** (0.952)	−1.25*** (5.64)	4.97*** (1.65)
Assets tangibility	−1.41*** (4.30)	−1.48** (7.52)	2.55*** (0.856)	0.038 (0.287)
Constant	−26.36*** (5.46)	−45.1*** (9.29)	−25.24*** (8.72*	−0.403 (2.60)
Wald test	42.24***	44.87***	8.76***	10.40***
AR (1)	−1.94	−2.16	−0.65	−1.02
AR (2)	−1.37	0.30	−0.42	0.95
Sargan Test	311.2	208.4	4.48	98.25
Observation	2,502	2,502	2,502	2,502

Level of significance in this study is indicated with “*” [***p < 0.01, **p < 0.05, and *p < 0.1].

**TABLE 5:** Firm size moderates the relationship between CEO duality and Chinese firm performance.

Variables	Model 5 (ROI)	Model 6 (ROE)	Model 7 (ROI)	Model 8 (ROE)
CEO duality	−1.58*** (0.469)	−2.11*** (0.754)	0.155** (0.082)	1.027*** (0.420)
Small firm	−1.32*** (0.539)	−2.24** (1.26)		
Large firm			0.503* (0.354)	−1.72*** (0.653)
Small firm*CEO duality	−2.81*** (0.801)	−2.99*** (0.908)		
Large firm*CEO duality			0.178* (0.115)	2.35*** (0.923)
Leverage	−14.16*** (2.30)	−15.87*** (2.85)	−2.54*** (0.514)	−2.97*** (0.681)
Growth	0.154** (0.083)	0.205*** (0.073)	0.085*** (0.035)	0.096** (0.044)
Firm size	−6.31*** (1.91)	−5.37 (5.20)	−1.74 (1.15)	9.55*** (2.42)
Assets tangibility	1.785*** (0.437)	3.17** (1.62)	0.754*** (0.328)	−1.61*** (0.639)
Constant	−15.5*** (4.34)	−28.7** (15.72)	−7.28*** (3.42)	17.54*** (6.69)
Wald test	27.75***	28.73***	28.44***	29.99***
AR (1)	−3.17	−3.31	−2.56	−2.90
AR (2)	−1.60	−0.81	−1.36	0.29
Sargan Test	43.66	41.15	1,773.48	587.41
Observation	2,502	2,502	2,502	2,502

Level of significance in this study is indicated with “*” [***p < 0.01, **p < 0.05, and *p < 0.1].

This study examined the robustness of the tests and used ROI and ROE to measure firm performance. It investigated the ROI performance measure and used ROI as the proxy in the first test. In the second phase, the ROE was applied as a proxy to measure performance to find a satisfactory level, as indicated in Tables 4, 5. These findings provided adequate results based on the two variables to measure firm performance. We applied the STATA software for analysis and to draw the results. Before incorporating the GMM, we performed some tests to analyze the validity of the instruments, that is, whether they are suitable for this analysis or not. First, we tested the variance inflation factor (VIF) approach to examine the multicollinearity issues among the chosen variables. The VIF test results indicated no multicollinearity problem in the variables of the selected model. The Wald test for heteroscedasticity was applied, and the results confirmed that no heteroscedasticity variables existed among the chosen variables. Third, a Sargan analysis was performed on the model to check the validity of the selected instruments and over-identifying restrictions. The Sargan analysis results showed that the instrument variables were valid for providing support for the chosen variables. Finally, we used a GMM method to solve the endogeneity issues between the error terms and variables. The outcomes of all the study tests revealed that the data provided adequate results and did not suffer from weak instruments.


(1) $$\Upsilon_{i,t}=\alpha_{1}+\beta_{1}\,{\text{X}}_{1\,i,t}+\gamma_{1}\,{\text{Z}}_{i,t}+\mu_{i,t}$$

$$\Upsilon_{i,t}=\alpha_{2}+\beta_{2}\,{\text{X}}_{1\,i,t}+\beta_{3}\,X_{2\,i,t}+\beta_{4}\,{\text{X}}_{1\,i,t}\times X_{2\,i,t}$$

(2) $$+\gamma_{2}\,{\text{Z}}_{i,t}+\mu_{i,t}$$

$$\Upsilon_{i,t}=\alpha_{3}+\beta_{5}\,{\text{X}}_{1\,i,t}+\beta_{6}\,X_{3\,i,t}+\beta_{7}\,X_{1\,i,t}\times X_{3\,i,t}$$

(3) $$+\gamma_{3}\,{\text{Z}}_{i,t}+\mu_{i,t}$$

$$\Upsilon_{i,t}=\alpha_{4}+\beta_{8}\,{\text{X}}_{1\,i,t}+\beta_{9}\,X_{4\,i,t}+\beta_{10}\,X_{1\,i,t}\times X_{4\,i,t}$$

(4) $$+\gamma_{4}\,{\text{Z}}_{i,t}+\mu_{i,t}$$

Here,

Υ_*i*,*t*_ =  refers to the firm performance of Business I at year t with two dependent variables, ROA and ROI, respectively;
*X*1_*i,t*_ =  represents CEO duality,
X2_*i,t*_ :  refers to a small firm,
X3_*i,t*_ :  refers to a large firm,
X4_*i,t*_ :  presents CSR I at year t,
Z_*i*,*t*_ :  indicates control variables,
α_*n*_ = 1, 2, 3, 4, 5 represents constant term,
γ_*n*_,β_*n*_ :η = 1,2,3,4,5,6,7  indicates constant to estimate respectively,
μ_*i,t*_ :  shows error terms for a firm I at year t.
Here, “I”
indicates firm performance (Chinese companies), with year “t” and ROA and ROI (two dependent variables).
It signifies the CEO duality influence of a firm,
It refers to a small firm,
It refers to a large firm,
It shows CSR practices for “I” at year “t,”
It indicates control variables,
While 1, 2, 3, 4, 5 represents the constant term,
It indicates constant to estimate respectively,
It shows error terms for the Chinese firms for “I” at year “t.”

## Results

### Empirical Results

Table 3 demonstrates the empirical analysis of the descriptive statistics of this study along with the VIF (variance inflation factor) of the dependent and independent variables of the study model based on “Panel A.” This study used two dependent variables, ROI and ROE. Panel A specifies that the average ROI value is 0.34 and that the standard deviation value is 0.605. The ROE value is 0.461, and the standard deviation is 0.985. Firm size and CEO duality have an average output of 10.09 and 0.258, and standard deviation values are 0.57 and 0.437. The capital growth indicates a mean value (M = 0.088) and a standard deviation (SD = 0.094). The firm advantage reveals an average value of 0.088. The asset tangibility shows mean value (M = 3), with a standard deviation (SD = 5.87), and the CSR an average value (CSR = 1.95), with a standard deviation (SD = 1.76).

“Panel A” specifies the descriptive statistics analysis. In a regression model, multicollinearity can result in higher standard error outcomes and make the inference difficult and biased. Accordingly, this study employed VIF analysis (variance inflation factor) to trace the multicollinearity issues. The study applied the VIF to confirm the absence of multicollinearity problems in this sample. Panel B examines the VIF analysis. The average values of the chosen variables are lower than 10, which confirms that the data are free from multicollinearity issues. Higher values of VIF might indicate that data suffer from multicollinearity problems (Hair et al., 2016, 2017). Table 3 shows the detailed VIF values related to Panel B. See Table 3 below.

### Hypotheses 1 and 2 Results

Table 4 indicates the results related to the stated hypotheses (H 1 and H 2) of this study and the coefficient estimates of variables to explore the direct relationship between CEO duality and the performance of Chinese firms with the moderating role of CSR in this relationship. Models 1 and 2 reveal the direct impact of CEO duality on firm performance with two different performance measurements, as ROI and ROE. Model 1 suggests that CEO duality impact is statistically significant and negative and that the coefficient value is ROI = −0.534. Besides, Model 2 displays that CEO duality is negative and substantial, with an ROE = −1.21 coefficient value. These results support hypothesis 1, which describes that CEO duality has a negative influence on firm performance. The study results designated that the effect of CEO duality exhibits a significant adverse relationship to both dependent variables ROI and ROE. The study results specify that CEO duality has a negative impact on Chinese firm performance. Models 3 and 4 exhibited that CSR activities moderate the relationship between the duality of CEOs and Chinese firm performance connection. This study created models 3 and 4 to explore how CSR practice plays a moderating role in the relationship between CEO duality and Chinese firm performance. Models 3 and 4 results show that the coefficient designated a fair value of 1.521 and 1.01 with performance measurements ROI and ROE, respectively. These outcomes specified that the CEO duality position exhibited a significant and positive influence on the performance of Chinese firms by adding CSR as a moderating variable. The positive impact of CSR on CEO duality and firm performance association confirmed the proposed hypothesis 2. Hypothesis 2 of this study stated that CSR practices moderate the relationship between the duality of CEOs and the performance of Chinese firms. The results advocate that CEO duality effects proved a substantial and positive relationship with Chinese performance measurements (ROI and ROE) through CSR moderating effects. The argument supported that this positive association is noticeable in Chinese firms with CSR activities. These findings confirm that the influence of CEO duality on business firm performance has a positive association with the effects of CSR and that the results of the study agreed with the past literature. As shown in Table 4 for further information.

### Hypothesis 3 Results

Table 5 presents the findings of this study. The results of this study indicate that CEO duality influences the performance of Chinese firms through the moderating role of firm size. Models 5 and 6 specify that CEO duality affects the performance of Chinese firms through the moderating role of small firms. Besides, model 5 designates that the coefficient value of CEO duality is ROI = −1.58 at a 1% significance level. Similarly, model 6 specifies that the coefficient value of CEO duality is −2.11, statistically significant at a 1% significance level. The study findings indicate that small firms have a significant and negative relationship with CEO duality and its impact on the performance of business firms, with performance measurements evidenced with ROI and ROE.

Models 7 and 8 display the connection between CEO duality and its impact on Chinese firm performance with the moderating effects of large firms. Model 7 presents the coefficient value of CEO duality to be 0.155 and stipulates a positive and statistically significant influence on business performance. Similarly, model 8 indicates a positive and considerable power of CEO duality coefficient with a value of 1.027. Both study models offer a positive coefficient and specify that large firms positively impact CEO duality influence on the performance of Chinese business firms (Raith, 2003). Refer to Table 5 below.

## Discussion

This study focused on investigating the relationship between CEO duality and the organizational performance of Chinese business firms through the moderating influence of firm size (large and small) and CSR. The findings of the study model are consistent with the literature (Hair et al., 2016; Lin et al., 2019; Madaleno and Vieira, 2020; Boakye et al., 2021; Javeed et al., 2021; Mittal and Garg, 2021). The first hypothesis that CEO duality negatively impacts the performance of Chinese firms was confirmed, as indicated in Table 4. Models 1 and 2 of the study reveal the coefficient values that directly influence the relationship between CEO duality and the performance of Chinese firms, with different performance measurements, such as ROI and ROE. Model 1 shows that CEO duality adversely impacts the performance of Chinese firms. A coefficient value of −0.534 confirms this relationship, as shown in Table 4. Model 2 indicates that CEO duality directly affects firm performance; the coefficient value of −1.21 proves this claim, as indicated in Table 5. Thus, the findings of this study support H1, which states that CEO duality adversely affects the performance of Chinese firms. CEOs with the dual role can take advantage of their position and work to benefit at the cost of firm performance. Therefore, the first hypothesis established some evidence about the negative relationship between CEO duality and the performance of Chinese firms. The agency theory supports this result. Firms typically allow CEOs with a dual role to act efficiently for their benefits (Fama and Jensen, 1983). Businesses can suffer from such concerns when the decisions of CEOs reveal private interests rather than shareholder interests. For instance, CEOs may decide to raise their particular interests and pursue discrete benefits at the expense of shareholders (Jensen, 1993). Thus, the association between the CEO duality effect and the performance of Chinese firms established a negative result and supported various past study outcomes (Yan and Lee, 2008; Tang, 2017; Naseem, 2020).

The second hypothesis posits that CEO duality has a positive effect on firm performance with CSR practices. Thus, H2 states that CEO duality impacts the performance of Chinese firms subject to a moderating CSR role. Hence, CSR practices moderate the relationship between CEO duality and the performance of business firms. Models 3 and 4 of this study show that CSR activities play a moderating role in the relationship between CEO duality and the performance of Chinese firms. Thus, this study created Models 3 and 4 to explore how CSR practices play a moderating role in the relationship between CEO duality and the performance of Chinese firms. Models 3 and 4 show the designated fair values of the coefficients (ROI = 1.521 and ROE = 1.01). The study outcomes indicate that CEO duality significantly influences the performance of Chinese firms by adding CSR as a moderating variable. Hence, these results confirm H2, as indicated in Table 4. The findings prove that CEO duality positively influences the performance of Chinese firms through the moderating role of CSR practices. The average increase in company performance due to CEO duality is more significant than the CSR activities of firms. These results indicate that CEOs can help enhance firm performance by adopting CSR activities and creating the right corporate image in the marketplace and public. Thus, CSR activities provide transparency and responsibility to their customers and create external pressure on CEOs to formulate a reasonable investment strategy (Javeed et al., 2020). In addition, CEOs with dual positions are better inspired to contribute to CSR activities and fulfill shareholder requirements, make their firms trustworthy with long-term survival, and build the right business image for the public (Adams et al., 2007; Islam and Deegan, 2008). Business firms of emerging countries have tried for proper representation in the marketplace. Besides, firms with CSR activities could increase their performance by building a well-reputed society (Javeed et al., 2020) and are supposed to be unique. Thus, this could lead to higher firm performance.

The third hypothesis states that firm size (small and large) moderates the relationship between CEO duality and Chinese firm performance. This study developed Models 5 and 6, which showed that CEO duality influences the business performance of firms subject to the moderating role of small Chinese firms. Model 5 designates the coefficient value of CEO duality (ROI = −1.58) at a 1% significant level. Besides, Model 6 specifies that the coefficient value of CEO duality is ROE = −2.11 and significant at 1% level, as indicated in Table 5. The findings show that small firms have a meaningful adverse relationship with CEO duality and impact on business performance. The performance measurements of ROI = −1.58 and ROE = −2.11 indicate a satisfactory result. This study recognized that small firms negatively affect the relationship between CEO duality and the performance of Chinese firms. These findings are in line with the literature results. A previous study has shown that small firms grow slowly and keep their executives relaxed, leading to lower profitability (Raith, 2003). Besides, small firms do not undertake publicly accountable activities because of their limited resources. Correspondingly, they use low-quality materials to manufacture goods and, thus, make lower profits. Small firms have few products and deal with low-profit margins, and do not reveal innovative behavior. Consequently, shareholders are not confident of investing in these firms.

Small firms have low resources and are unable to compete in competitive and uncontrolled business environments. This study supports the proposed hypotheses and shows that firm size can change the CEO duality and firm performance relationship. Besides, the study shows that large firms positively affect the relationship between CEO duality and the performance of Chinese firms. It is because large firms have more resources and a well-organized structure. They can force executives to carry out business activities with clear intent to make the business more profitable because their executives have fewer chances to pursue their benefits. Besides, large firms require a differentiated strategy for higher market share to maximize profits. The well-organized structure of large firms enables their CEOs to take fast and effective short-term decisions, which is vital for the performance of large firms. Large firms can enjoy the benefits of CEO duality because of their unparalleled environmental efficiency. Having a single decision-maker or manager can be vital for large business firms in dynamic environments. Environmental progressive states to the instability of the situation, and it plays a role in the connection of CEO duality influence on business performance.

Hypothesis 3 (a) proposes a positive relationship between CEO duality and the performance of large-sized Chinese firms. This study created Models 7 and 8, and their findings (ROI = 0.155 and ROE = 1.027) indicate that CEO duality impacts the performance of large Chinese firms. Model 7 presents the coefficient value of CEO duality (ROI = 0.155) and stipulates a positive and statistically significant effect on the business performance of Chinese firms. Similarly, Model 8 indicates a positive and considerable CEO duality coefficient value (ROE = 1.027). Both study models offer a positive coefficient and show that large firms positively impact the relationship between CEO duality and the performance of Chinese business firms. These results support the proposed hypotheses, as indicated in Table 5. Thus, our study findings support the proposed hypotheses and show that CEO duality influences firm performance through the moderating role of firm size. This article adds to the theoretical discussions related to the duality theories of CEOs, which suggest that dual roles of CEOs of firms are helpful in achieving the better performance of business firms (Dalton et al., 1998; Mahmood et al., 2019).

Future studies can explore the current crisis posed by the coronavirus disease-2019 (COVID-19) pandemic and examine various factors that can help improve firm performance (Akhtar et al., 2020a,b; Ali et al., 2020; Anser et al., 2020c; Usman et al., 2021). For instance, media communication, crisis management, and leadership can influence firm performance (Shuja et al., 2020a; Yoosefi et al., 2020b; Azizi et al., 2021; Su et al., 2021a; Wang et al., 2021). Similarly, marketing strategies, innovation, environmental factors, entrepreneurial resources, and social good can influence the behavior of consumers, as the health security of company employees is an essential factor for firm growth (Usman et al., 2018, 2019; Anser et al., 2020a,c; Shuja et al., 2020b; Abbas et al., 2021; Local Burden of Disease HIV Collaborators, 2021; Maqsood et al., 2021; Su et al., 2021b). Adopting preventive measures is very important for the safety and health security of employees, affecting the productivity of employees considerably for better firm performance (Anser et al., 2020b,d; Azadi et al., 2021). Business firms need to formulate strategies to combat the adverse effects of COVID-19 on the mental health of their employees, as the pandemic has affected the domestic lives of workers (Yoosefi et al., 2020a; Aman et al., 2021; Taufik et al., 2021). Firms dealing with the electricity consumption of the industrial sector, electricity price, and demand have faced tremendous pressure to provide a smooth power supply (Wang et al., 2020; Raza et al., 2021; Wan et al., 2021). Thus, the ongoing pandemic has seriously affected the performance of firms in various sectors worldwide (Abbasi et al., 2021; Hu and Zhang, 2021; Jin et al., 2021).

## Conclusion

This research emphasized examining the association between the duality of CEO and organizational performance of Chinese business firms through the moderating effects of firm size (large and small) and CSR. The study results are in line and consistent with the body of past scientific literature. This study identified potential literature gaps to explore the relationship between the duality of CEOs and business firms operating in China. The study examined a moderating effect of firm size (small and large) and CSR on this relationship. According to the best of this research investigation, the literature did not evidence this relationship that CEO duality influences the performance of Chinese firms with the moderating role of firm size and CSR. This study employed the GMM model for analysis purposes, as many past studies have recommended this methodology. The results of this study model are consistent with the past literature. The study findings, as indicated in Tables 4, 5, have supported all the proposed hypotheses. Accordingly, the study results confirmed the hypotheses and demonstrated that CEO duality influences firm performance through the moderating role of firm size and CSR practices. This study adds its new contribution to the existing body of literature based on the performance of Chinese business firms, with the effective influence of the CEO being twofold.

This study model observes how firm size (small and large firms) and CSR activities moderate the relationship between CEO duality and business performance. Accordingly, this research offers a valuable and insightful contribution to the scientific literature based on the relationship between CEO duality and Chinese firm performance. The model analyzes a moderating effect of CSR practices and firm size in this connection. This research employed data of Chinese business organizations to study the relationship between CEO duality and company business performance. This research provides evidence from China, and the outcomes of this study describe that CEO duality negatively influences firm performance. Besides, the findings exhibited that CSR positively moderated the relationship between CEO duality and firm performance. Correspondingly, the results specified that small firms revealed a negative association between the relationships under investigation. However, large and sizeable Chinese business firms positively moderate the connection between CEO duality and better business performance. Hence, CEO dual roles can enjoy the benefits of their twofold roles in large companies practicing CSR because of their unparalleled environment efficiency. The research findings stated that institutional mechanisms play an indispensable role in achieving healthier business performance. Implementing this study results in the research field related to firm performance and corporate governance literature would be beneficial to gain better Chinese firm performance.

## Theoretical Implications

This research model provides significant contributions to the scientific literature of the dual role of the CEO and its influence on firm performance with the moderating impact of Chinese business firms and CSR practices. This study model has extended the past research and provided a comprehensive and better view of the moderating effects of firm size and CSR practices between the relationship of CEO dual roles and firm performance. The findings designated and theorized that Chinese business firms and CEO duality indicated a positive relationship and that CSR has positively moderated this relationship to attain better business performance. The past literature has not documented this relationship, and this study filled the identified gap and contributed to the literature. Accordingly, developing a comprehensive model by employing the datasets of listed Chinese companies and implementing the GMM technique to draw the results, the model describes that the results are comprehensive and robust. It explores how CSR and firm size have moderated the relationship between CEO dual functions and better business performance. This innovative model has examined a new framework, and past research has not explored this direct relationship through moderating effects as indicated in this study. This study robustly proves the channel, transmitting the influence of CSR practices and firm size (small/large) with an indispensable corporate reputation to attain good business performance. This research is the first that debated and documented that the CEO dual position significantly plays a vital role in driving CSR practice effectiveness. This model provides valuable insights into the crucial role of CEO duality to moderate firm size and CSR practices. Past research has not investigated this relationship and effect between CEO duality and Chinese firm performance. This study laid foundations to develop measurement to examine the dual role of CEO through double moderating effects to check firm performance.

### Practical Implications

The findings of this study recommend some future implications to describe the benefits of the dual role of CEO on Chinese firm performance with the moderating influence of firm size (small/large) and CSR practices. First, executives need to consider the demands of their stakeholders and develop a robust approach to solving their problems. For instance, a CEO can increase the profits of a firm by improving CSR activities and engaging employees and societies. Second, this study encourages investors to play an active role in investing in large business firms to exploit more profits. Besides, the findings of this research study acknowledged that firm size moderated the impact on the relationship between CEO duality and Chinese firm performance. It might lead key shareholders to invest in large firms to take advantage of CEO dual positions. Third, the most critical two-leadership positions are not necessary/must for healthier business performance, even though the joint leadership position is beneficial for the firm and helps develop firm values due to closer relationships among managers and shareholders. CEO duality is not necessarily for adverse effects on firm performance. This study also added to the theoretical discussions of CEO duality theories, which proposed that CEO dual role leads to superior firm performance.

### Limitations of the Study and Agenda for Future Research

This study has some limitations like any empirical research. First, the study datasets reported on a sample of Chinese business firms (listed companies), and significant limitations refer to a unique organized business environment for Chinese firms. However, it is the second-biggest global market. For instance, publicly available information influences the market, and it will manifest in share prices. Thus, the findings of this research model might not offer generalizability for other regions and countries. However, studies from multiple contexts are relatively rare. Conversely, the limitations suggest a potential for future studies and might help understand the connection of CEO duality and firm performance through double moderating effects with different variables. Besides, this research only discussed the moderating role of firm size (small and large) and CSR on the relationship of CEO dual roles and firm performance. It did not comprise other relevant firm strategies (ownership structure) to describe the relationship between CEO duality and firm performance. Hence, future studies can test these strategies and add some other mediators or moderator variables. This research model recommends other potential moderators, such as ownership structure, firm age, and financing configuration, by selecting some other economy for future studies to explore compelling results for business managers, CEOs, and other stakeholders.

## Data Availability Statement

The original contributions presented in the study are included in the article/supplementary material, further inquiries can be directed to the corresponding author/s.

## Ethics Statement

The Ethics Committee of the School of Management, Harbin Institute of Technology (HIT) reviewed and approved the research project. The participants provided their written, informed consent to participate in this study.

## Author Contributions

RM and JA conceptualized the idea, contributed to the study design, completed the entire manuscript, including introduction, literature, discussion, conclusion, and edited the original manuscript before submission. DH reviewed and approved the final edited version and approved the submitted version. SÁ-O and MS significantly helped and provided major contributions in revising this manuscript and also contributed to resources to make this manuscript possible. All the authors reviewed and approved the final edited version and approved the submitted version.

## Conflict of Interest

The authors declare that the research was conducted in the absence of any commercial or financial relationships that could be construed as a potential conflict of interest.

## Publisher’s Note

All claims expressed in this article are solely those of the authors and do not necessarily represent those of their affiliated organizations, or those of the publisher, the editors and the reviewers. Any product that may be evaluated in this article, or claim that may be made by its manufacturer, is not guaranteed or endorsed by the publisher.
